# Money, Medicine, and Motherhood: Developing a Cash Transfer for Pregnant Women with HIV in Rural Haiti

**DOI:** 10.1007/s10461-025-04933-2

**Published:** 2025-11-06

**Authors:** Aaron Richterman, Sindy Desire, Wendel Blaise, Elyse Derose, Alexis Moise, Lisa P. Ward, Christophe Millien, Harsha Thirumurthy, Florence Momplaisir, Louise C. Ivers

**Affiliations:** 1 Division of Infectious Diseases, Department of Medicine, University of Pennsylvania, Philadelphia, PA USA; 2 Department of Medical Ethics and Health Policy, University of Pennsylvania, Philadelphia, PA USA; 3 Penn Leonard Davis Institute of Health Economics, Philadelphia, PA USA; 4 Health Equity International/St. Boniface Hospital, Fond-des-Blancs, Haiti; 5 Mirebalais University Hospital, Zanmi Lasante, Mirebalais, Haiti; 6 Center for Global Health, Massachusetts General Hospital, Boston, MA USA; 7 Department of Global Health and Social Medicine, Harvard Medical School, Boston, MA USA

**Keywords:** HIV, Pregnancy, Cash transfers, Poverty, Qualitative research

## Abstract

Improving perinatal engagement in HIV care is critical to prevent disease progression and transmission. In Haiti and other low-resource settings, poverty contributes substantially to poor perinatal HIV outcomes. Cash transfers are a promising intervention that may alleviate poverty and improve perinatal HIV outcomes through economic and psychological pathways. To inform the design of an unconditional cash transfer intervention, we conducted semi-structured interviews with 20 pregnant and postpartum women with HIV receiving care at St. Boniface Hospital in rural Haiti 12/2023-5/2024. Using an interview guide with open-ended questions, we explored barriers and facilitators to HIV care around the time of birth and perspectives on and preferred features of a potential unconditional cash transfer. Interviews were conducted in Haitian Creole, transcribed and translated into English, and analyzed thematically using a codebook developed through deductive and inductive coding. Participants (median age 24 years; 50% pregnant) described barriers including antiretroviral therapy side effects, particularly when taken without food, interpersonal stigma, poverty, and food insecurity. Facilitators included motivation to protect the child’s health, understanding treatment benefits, transportation reimbursement, and social support. Participants viewed cash transfers as a promising way to address poverty-related barriers, reporting they would use funds for household needs, child expenses, and small business activities. Preferred transfer sizes varied, as did preferences about delivery, timing, and frequency. Findings underscore poverty’s central role in shaping perinatal HIV care engagement and support the potential of unconditional cash transfers to improve outcomes.

**Supplementary Information:**

The online version contains supplementary material available at 10.1007/s10461-025-04933-2.

## Introduction

Improving short- and long-term virological outcomes for pregnant women with HIV is essential to preventing disease progression, drug resistance, transmission to partners, and perinatal transmission during current and future pregnancies. Although antenatal care engagement is relatively high in many settings, global rates of postpartum clinic attendance, antiretroviral therapy adherence, and viral suppression for women with HIV remain unacceptably low despite longstanding efforts to prevent vertical transmission through engagement in health care [1–10]. 

Poverty is a widely recognized upstream driver of poor engagement in HIV care during and after pregnancy [5, 9]. This problem is especially pressing in Haiti, where 25% of the population lives in extreme poverty. Between 2011 and 2017, pregnant and breastfeeding individuals accounted for 30% of people with HIV initiating antiretroviral therapy [11], yet fewer than half were retained in care 12 months after starting treatment [1, 12–14]. 

Emerging evidence suggests that poverty worsens health outcomes not only through *economic* mechanisms but also through *psychological* mechanisms. These psychological mechanisms may include reductions in mental bandwidth (the mental resources used for decision-making) and increases in future discounting (the tendency to undervalue future outcomes) [15–17]. These constraints may be particularly acute during pregnancy and in the context of HIV [18], due to additional physical, medical, social, and financial demands, as well as elevated risk of depression [19–23]. Among low-income women in the US, we have previously shown that living with HIV was associated with lower mental bandwidth and that, among people with HIV, greater mental bandwidth was associated with viral suppression [18]. 

Consequently, anti-poverty interventions like cash transfers that target low-income pregnant women with HIV may be particularly effective in promoting healthy behaviors and durably improving health outcomes [24]. While cash transfer programs have shown benefits across a range of outcomes [25–27], most HIV-related studies have focused on conditional cash transfers, which require specific behaviors for disbursement (e.g., school attendance for children) [28, 29]. Our prior research suggests that both unconditional and conditional transfers similarly improve some HIV-related and general health outcomes [26, 27]. However, few studies have examined whether unconditional transfers—which are simpler to administer and more directly address poverty—can specifically improve outcomes for pregnant women with HIV.

To understand the potential role of unconditional cash transfers in improving outcomes for women during pregnancy and the postpartum period, it is important to first evaluate the context in which such an intervention would be implemented. In this formative study, we conducted qualitative interviews with pregnant and postpartum women with HIV in Haiti to identify facilitators and barriers to HIV care engagement and to gather perspectives on the design of a potential unconditional cash transfer intervention to improve HIV-related engagement in care.

## Methods

We conducted individual semi-structured interviews of pregnant and postpartum women with HIV at St. Boniface Hospital in rural Haiti. We reported this study according to the Consolidated Criteria for Reporting Qualitative Research [30]. The Institutional Review Boards at the University of Pennsylvania and St. Boniface Hospital approved the study. All participants provided written informed consent.

### Setting

The study took place at St. Boniface Hospital, a 184-bed facility and network of community health programs operated by Health Equity International in Fond-des-Blancs, Haiti. Fond-des-Blancs is a town in Haiti’s Southern peninsula, a mostly rural region of 2.3 million people [31]. At the time of the study, St. Boniface Hospital employed two full-time and two part-time obstetricians, and two full-time and two part-time pediatricians. Annually, the hospital has over 10,000 prenatal visits with over 3,000 deliveries. About 750 women with HIV are followed longitudinally through the hospital’s HIV program, and around 100 women with HIV deliver at the hospital each year. The viral suppression rate among pregnant women with HIV at St. Boniface is approximately 50%, consistent with similar settings [3, 4, 32]. There was significant geopolitical instability, gang-related violence, and economic hardship in Haiti during the study.

### Participants

Eligibility criteria included: (1) pregnant or < 6 months postpartum, (2) living with HIV, and (3) cisgender female. Potential participants were identified by study staff in HIV and perinatal clinics at St. Boniface Hospital. Interested individuals provided informed consent in Haitian Creole, basic quantitative data (age, pregnancy status, number of children), and scheduled an interview. We used purposive sampling to include both pregnant and postpartum participants, aiming for a minimum of 20 participants [33, 34]. We had a planned sample size of at least 20 participants, with the final sample size determined after saturation of key themes. No participants withdrew from the study.

### Interview Procedures

We developed a semi-structured interview guide consisting of questions in two sections (see interview guide in Supplementary Materials): the first section broadly and in an open-ended fashion addressed current facilitators and barriers to sustained engagement in HIV care (with separate questions about clinical follow-up and medication adherence, although ultimately participants did not substantially distinguish between these processes), while the second section explored perspectives on a potential unconditional cash transfer intervention with the objective of improving engagement in HIV care. In addition to general impressions, we elicited participants’ views on optimal intervention characteristics including amount, timing, frequency, and payment mechanism. The interview guide was informed by the Consolidated Framework for Implementation Research (CFIR), ensuring relevance to future implementation considerations [35]. Individual interviews were conducted in-person, in a private office, and in Haitian Creole by a member of the study staff trained in qualitative research (ED). All interviews were audio recorded, transcribed verbatim, and translated into English.

### Data Analysis

We summarized quantitative data using descriptive statistics. For qualitative data, we applied an integrated analysis approach, combining deductive and inductive thematic analysis [36]. Initially, we developed a codebook based on predetermined domains related to facilitators and barriers to HIV care around the time of birth, as well as characteristics of unconditional cash transfer interventions. As analysis progressed, the codebook was refined iteratively to incorporate emergent themes. Two researchers (AR and SD) coded the transcripts, coding the first two together by consensus, then the next two independently but subsequently reviewed together to ensure consensus, and then the remainder with 20% double-coding to ensure consensus [37]. Disagreements were resolved by consensus in discussion with the study team. We identified representative quotes to illustrate key themes. NVIVO software (Lumivero; Version 15) was used for qualitative data management and analysis.

### Reflexivity Statement

The research team comprised physician-scientists and public health researchers with expertise in HIV, maternal health, and poverty-related interventions. Investigators were based in the United States and Haiti and did not share the lived experience of being pregnant or postpartum with HIV in rural Haiti. To ensure cultural relevance and sensitivity, Haitian team members provided input on the study design, recruitment approach, and interview guides. We acknowledge that our professional roles, educational backgrounds, and positionality as researchers working in a low-income setting may have influenced the framing of interview questions and interpretation of findings. We sought to mitigate these influences through iterative team discussions and consensus-building across a multidisciplinary, cross-cultural research team.

## Results

We enrolled 20 pregnant and postpartum women with HIV between December 2023 and May 2024. Half of the participants were pregnant at the time of enrollment, and their median age was 24 years (IQR 21 to 28.5). Participants had a median of one child at the time of the interview (IQR 1 to 3).

### Facilitators of Engagement in HIV Care

All participants were receiving antiretroviral therapy and consistently expressed high motivation to continue treatment. Nearly all participants identified protecting their child’s health as the primary short-term motivator for maintaining HIV care engagement. One participant shared, “*especially while I’m pregnant*,* I will always take my medication… I will always manage all the means to do it*,* just so that the child doesn’t get infected because I don’t want him to get infected*” (Participant 6, pregnant, no children). Preserving their own health, on the other hand, was described as an important long-term motivator. As one stated: “*If I don’t take these medications anymore*,* it will get worse for me. I will get sick*,* have diarrhea*,* and my body will be full of bumps*” (Participant 1, pregnant, four children).

About half of the participants reported that at least one other household member was aware of their HIV diagnosis. Among these participants, family support was cited as a significant facilitator of antiretroviral therapy adherence. One participant explained, “*only my mother knows*,* she always helps me watch the time. She always helps me stay on track and when we follow up*,* she’s always available*” (Participant 13, postpartum, one child). Another emphasized, “*[my mother] always gives me advice to take [the medications]*,* and she always tries to get me to take the medicine early [in the day]*” (Patient 11, pregnant, one child).

Most participants highlighted the supportive care provided by St. Boniface Hospital as critical to their ongoing engagement in HIV care. In particular, many participants lived hours away from the hospital, requiring travel by motorcycle, and the hospital routinely covered transportation costs. One participant described, “*when I come to St. Boniface Hospital*,* they give me a lot of courage… many times when I come to the hospital*,* I don’t have money to come… and they always pay for my round trip*” (Participant 6, pregnant, no children).

Transportation reimbursement was the primary material support provided by the hospital. A few participants reported occasionally receiving other forms of material support from the hospital, such as food or financial assistance. One participant who received food aid shared, “*it helped me a lot because I used to buy very expensive small cups of rice*” (Patient 12, postpartum, three children). Another participant emphasized the infrequent nature of this material support when she shared, “*I once received food from them but never again*” (Participant 20, pregnant, no children).

### Barriers to Engagement in HIV Care

Participants described several key barriers to engagement in HIV care around the time of birth. Side effects of antiretroviral therapy, particularly nausea and dizziness when medications were taken without food, were frequently reported. One participant explained, “*whenever I take the medication without food*,* it causes me a lot of problems*,* sometimes I throw up*,* sometimes I get dizzy*,* I don’t feel good at all*” (Participant 6, pregnant, no children).

Many participants highlighted that side effects of antiretroviral therapy overlapped significantly with symptoms associated with hunger and pregnancy, emphasizing that food insecurity often prevented consistent antiretroviral therapy adherence. Outside of food insecurity, lack of resources more generally was seen as a barrier especially for being able to attend to clinical follow-up—one participant stated, “*[a lack of resources] does not stop me completely*,* but I do not come every time*” (Participant 17, pregnant, one child). Despite these challenges, participants remained motivated to take their medication, as described by one postpartum participant: “*what can affect me taking them is the problem of not having the means… you know how life is right now. No money. Although things are like this*,* even when I don’t have food*,* I still try to take the medicine*” (Participant 7, postpartum, two children).

Additionally, interpersonal and community stigma were frequently cited as a significant barrier, especially among participants who had not disclosed their HIV diagnosis to household members. A participant commented, “*they look at my appearance… they think I can harm them*” (Participant 6, pregnant, no children). The consequences of stigma could be severe, as illustrated by another participant’s experience: “*in the place where I was staying*,* someone brought me medicine. Since the person didn’t find me*,* they left the medicine*,* and said it was for me to give to my grandmother. However*,* others understood and*,* as a result*,* I was kicked out of the house*” (Participant 2, pregnant, one child).

### Unconditional Cash Transfer Characteristics

Several themes emerged when participants discussed preferences for a hypothetical unconditional cash transfer intervention for pregnant women with HIV. About half of the participants indicated that they would use the cash to address immediate basic needs, particularly those related to caring for their children. One participant explained, “*If I was going to buy a cup*,* now I will have enough to [also] buy a bar of soap*” (Participant 9, postpartum, four children). Other participants described plans to invest the cash in small household business activities. As one participant shared, “*I could make the money grow. Regardless of the amount*,* small money leads to big money*” (Participant 2, pregnant, one child). Participants who would use the cash for immediate basic needs generally preferred to receive the cash in installments, whereas those who intended to invest in small household business activities said they would prefer receiving a lump-sum payment. While most participants focused on how a cash transfer would alleviate poverty-related needs (and these basic needs were previously identified as barriers to engagement in HIV care), a few participants also directly connected a potential cash transfer with improvement engagement in HIV care. A participant shared, “*if I had enough money for that*,* there are times I would not have … spent several days without taking my medication*” (Participant 6, pregnant, no children). Another said, “*if it allowed me to have food to eat so I can take the medication*,* I would not have to refuse to take it*” (Participant 19, not pregnant, two children).

When asked about the total amount needed for an unconditional cash transfer intervention to meaningfully impact their lives, participants were hesitant to suggest specific amounts. Ultimately, the median total suggested was equivalent to USD $190 (IQR $114 to $314), with individual responses ranging from the equivalent of $19 to $1000. One participant summarized the potential impact as follows, “*If I had [USD $114]*,* I know that it will not change my life forever*,* but it will change my life for a while*” (Participant 10, postpartum, one child).

When asked about timing, if the transfer could only be given either before or after delivery, approximately two-thirds of participants expressed a preference for receiving it postpartum. One participant explained this preference: “*because after I give birth*,* I will be stuck inside. Because I’m the one who goes out to hustle*,* when I’m inside I won’t be able to find anything to give someone. With children also going to school*,* it’s just me. Everything is on me. It would be better for me after I give birth. Like when I’m inside*,* and if [the children] needed something*,* they would say*,* ‘Mom can you give me 25 gourdes [Haitian currency].’ And this [the cash transfer] would let me get it and give it to them*,* without people knowing my business”* (Participant 1, pregnant, four children). However, participants also noted potential benefits of receiving cash before delivery, particularly to help prepare for the new child. As one participant stated, “*if I received it while I was pregnant*,* it would be good for me because I’m going to have a baby and there are a lot of things I don’t have yet*” (Participant 6, pregnant, no children). These preferences did not necessarily align with participant preferences to receive the cash transfer in installments or as a lump sum.

Regarding the preferred payment mechanism, some participants preferred mobile phone payments, while others preferred cash disbursement directly from the hospital cashier. Preferences were generally influenced by participants’ circumstances, particularly concerns around confidentiality and fears of inadvertent disclosure of HIV status or that the participant was receiving a cash benefit. One participant highlighted these confidentiality concerns clearly: “*because if I send it to someone’s [cash app]*,* now they would see who sent it*,* which hospital sent it*,* the person will be suspicious*,* they will dig*,* dig*,* dig to find out how why the hospital is helping that person*” (Participant 18, pregnant, four children).

## Discussion

This qualitative study explored barriers and facilitators to engagement in HIV care among pregnant and postpartum women living with HIV in rural Haiti, as well as their perspectives on a potential unconditional cash transfer intervention. Reported barriers included antiretroviral therapy side effects (particularly when not having food), interpersonal and community stigma, poverty, and food insecurity. Facilitators included strong motivation to protect their child’s health, an understanding of the benefits of antiretroviral therapy, transportation reimbursement from the hospital, and social or family support when disclosure was possible. While not all participants directly linked a hypothetical unconditional cash transfer to better HIV outcomes, many said it would address food insecurity and lack of resources, which were among their major barriers to engagement in care. This supports our hypothesis that an unconditional cash transfer intervention may help improve HIV outcomes around the time of birth. Notably, several participants also directly linked the idea of cash support to improved engagement in care, highlighting its potential as a poverty-alleviating strategy with clinical relevance.

Our results align with existing literature that identifies multiple barriers to HIV care engagement around the time of birth across diverse settings. These include antiretroviral therapy side effects, pill burden, economic hardship, stigma, physical symptoms, mental health challenges, lack of social support, and fear of disclosure [3, 4, 9, 38, 39]. 

The cash transfer amounts proposed by the participants were about 10% of per capita gross domestic product in Haiti, which is similar to the median size of government-led cash transfer programs we included in large multi-country evaluations of low- and middle-income countries [26, 27]. Participants reported they would use an unconditional cash transfer either to meet immediate needs—particularly related to their children—or to invest in small household business activities. Preferences for disbursement reflected this divide: those focused on meeting day-to-day needs tended to favor receiving the cash in installments, while those interested in investing in income-generating activities preferred a lump sum. These differing preferences reflect the tradeoff between consumption smoothing and capital investment, an important consideration for intervention design that has been noted in prior studies of unconditional cash transfers [40]. An unconditional cash transfer aimed at alleviating basic needs around pregnancy may be most effective if delivered in regular installments.

Although many pregnancy-related benefits are disbursed postpartum (e.g., cash transfers for families with young children) [27], participants recognized the value of support before and after delivery. Preferences for timing were often driven by practical concerns, such as the inability to work immediately after childbirth or the need to prepare for the arrival of a baby. These perspectives support growing policy interest in extending postpartum benefits to span both the prenatal and postpartum periods [41, 42]. 

Confidentiality also emerged as a critical consideration in cash transfer delivery. This may especially be the case in small, rural communities where social networks are close-knit and stigma remains a concern for people with HIV. Though not explicitly mentioned, the severe geopolitical instability in Haiti at the time of data collection may have further heightened these concerns. These findings underscore the importance of offering discreet, participant-centered payment options and protecting privacy in implementation [43]. 

The primary limitation of this study was that all participants were recruited from perinatal clinics and were already engaged in some level of HIV care. As such, the sample may not represent the experiences of individuals who are less engaged (or not engaged) in care. However, the population we studied has a high attrition rate (50% at 12 months) and reflects the primary target for future interventions aimed at sustaining retention and adherence during the critical perinatal period. An additional limitation is that we did not address broader questions of affordability or scalability of cash transfer interventions within Haiti. These are important considerations for policy implementation. However, it is worth noting that unconditional cash transfers have been deployed at scale in a number of low- and middle-income countries, often with the support of donors and governments. Our findings provide early-stage, contextual insights that can inform the development and evaluation of such an intervention for pregnant women with HIV. Lastly, while we elicited participant perspectives on the importance of confidentiality, we did not specifically address the potential concern of partner-associated risks (e.g., intimate partner violence) that might result from cash transfers directed to women. However, nearly all prior studies of cash transfers have found reductions in the risk of physical harm, gender-based violence, and intimate partner violence, thought to result from increased women’s empowerment within the household [44, 45]. 

## Conclusion

Our findings highlight the complex interplay of structural, psychosocial, and interpersonal factors that influence engagement in HIV care among pregnant and postpartum women in rural Haiti. Participants identified multiple barriers—many rooted in poverty and exacerbated by food insecurity and stigma—and emphasized the importance of addressing child-related needs. An unconditional cash transfer intervention, if designed with participant priorities in mind, may offer a promising approach to support engagement in care and improve maternal and child health outcomes in this high-need population.

## Supplementary Information

Below is the link to the electronic supplementary material.

Supplementary Material 1
