# Supplementary material for: Money, Medicine, and Motherhood: Developing a Cash Transfer for Pregnant Women with HIV in Rural Haiti
Source: AIDS Behav. 2025 Nov 6;30(4):962–8. doi: 10.1007/s10461-025-04933-2 (PMC13076426; doi:10.1007/s10461-025-04933-2)
Supplement: Supplementary file 1 — Supplementary Material 1 [file 10461_2025_4933_MOESM1_ESM.docx]

**Patient Interview Guide (by Domain)**

*Introduction*

I will now be asking you some questions about different factors that may affect your health. I will also ask you some questions about a cash intervention we are developing. You will not receive this cash intervention as part of this study. If we do conduct a study of this intervention in the future and you are eligible for the study, you will be offered the chance to participate. As a reminder, your participation is entirely voluntary and you may end the discussion at any time. We will be recording this session, but the recording will be deleted after it has been transcribed.

*Part 1:*

*General:*

- Tell me about your experience taking medications for HIV.
- What things in your life affect your medication adherence?

*Economic:*

- How do money or food concerns impact how you take ART?

*Social/Interpersonal*

- How do other people in your household affect your ability to stay healthy?
- Who in your household is aware of your HIV diagnosis? In what ways are they supportive or unsupportive? How does your relationship with your household affect your medication adherence?

*Biomedical*

- Tell me about whether and how HIV medicine helps you.
- What will happen if you stop taking HIV medicine?

*Pregnancy-related*

- How do you feel having a new baby changes your thinking about coming to HIV clinic and taking HIV medicines?

*Structural*

- What are positive/negative aspects of the care you receive at this hospital?
- How does distance from the hospital affect how you take ART?
- Do you receive social support as part of your medical care; if so, in what ways does this fit and not fit with your needs?

*Part 2:*

*General:*

- If pregnant women with HIV were to receive a regular cash benefit, can you describe practically how this would work?
- What logistical challenges do you foresee?
- Would you have any privacy or confidentiality concerns about receiving such a benefit? How could these be minimized?

*Amount:*

- What would be the minimal amount of a cash benefit that would make a difference in your day-to-day life?
- Specifically, how would different cash benefit amounts change your life, what specific burdens would be relieved?

*Timing:*

- If you could only get a cash benefit either during pregnancy or in the months after pregnancy, which would be more helpful and why?

*Frequency:*

- Is it better to receive a cash benefit during pregnancy as a lump sum, or as a regular payment?

*Duration:*

- If a cash benefit during pregnancy could only be given for a limited amount of time, what is the minimum amount of time that would make a meaningful difference in your life?

*Payment Mechanism:*

- In what form should a potential cash benefits be given to pregnant patients? For example, direct cash from a clinic or community health worker, or a mobile bank transfer? Please be as specific as possible.

*Wrap up:*

- Age
- Pregnant or post-partum
- Number of children

We have now concluded the interview portion of the study. Thank you for your participation.

**Gid Entèvyou Pou Pasyan yo**

*Entwodiksyon*

Kounye a, m ap poze w plizyè kesyon sou faktè ki ka afekte sante w. M ap mande w tou kesyon sou karakteristik potansyèl entèvansyon transfè lajan. M vle fè ou sonje ou pap resevwa entèvensyon sa a nan etid sa. Si nou fè yon etid sou entèvansyon sa a nan tan kap vini an, e ou enterese patisipe, n ap ofri w opòtinite pou w patisipe si w kalifye. Patisipasyon w se konplètman volontè, epi ou ka fini entèvyou a nenpò ki lè. M pral anrejistre odyo entèvyou a. Nou pral transkri odyo anrejistreman, epi n ap kraze odyo anrejistreman yo. M mande w pa di ni non w ni lòt non ni kote yo ni enfòmasyon yo ki ka idantifye tèt w oubyen yon lòt moun.

*Pati 1:*

*Jeneral:*

- Pale m de eksperyans ou fè lè w bwè medikaman pou VIH?
- Ki bagay nan lavi ou ki ta ka afekte kapasite w pou w bwè medikaman yo chak jou?
  - (Swiv kesyon yo):
  - Kisa ki fè li pi fasil?
  - Kisa ki fè li pi difisil?

*Ekonomic:*

- Kijan enkyetid lajan oubyen manje afekte fason ou ka bwè medikaman ou yo?

*Sosyal/Entèpèsonèl:*

- Kijan moun lakay w afekte kapasite w pou w rete an sante?
- Kiyès moun lakay w ki konnen w gen VIH?
  - (Swiv kesyon yo):
  - Nan ki fason yo sipòte w?
  - Nan ki fason yo pa sipòte w?
  - Kijan relasyon ou ak moun lakay ou afekte kapasite w pou w bwè medikaman yo chak jou?

*Medikal:*

- Di m si medikaman kont VIH ede w? Si wi, kijan?
- Ki sa kap fèt si w sispann bwè medikaman kont VIH?

*Ki gen rapò ak gwosès:*

- Kijan akouchman ou, ak fè yon nouvo bebe ka afekte kapasite w pou w swiv nan klinik VIH?
- Ki jan akouchman ou, ak fè yon nouvo tibebe afekte kapasite w pou w bwè medikaman kont VIH chak jou?

*Estriktirèl:*

- Ki sa ki bon nan swen sante ou resevwa nan Lopital Sen Bonifas?
- Ki sa ki mal nan swen sante ou resevwa non Lopital Sen Bonifas?
- Ki jan distans ak lopital la afekte kapasite w pou w vin pran medikaman kont VIH oubyen resevwa yo chak jou?
- Eskè w pran nan men Lopital Sen Bonifas sipò materyel?
  - Si wi, ki jan sipò sa a satisfè sa w bezwen?
  - Si wi, ki jan sipò sa a pa satisfè sa w bezwen?

*Pati 2:*

*Jeneral:*

- Si fanm ansent ki gen maladi VIH ta resevwa yon benefis lajan regliyeman, dekri pou mwen kijan sa a ta fèt pratikman? Tanpri bay otan detay ke ou kapab.
- Eskè ou prevwa okenn defi pou yon benefis konsa?
- Eskè w gen okenn enkyetid de konfidansyalite sou resevwa yon benefis konsa?
  - Si wi, ki jan yo ta ka minimize enkyetid sa yo?

*Kantite:*

- Ki kantite lajan ki ta yon minimòm benefis pou fè yon diferans nan lavi w chak jou ?
- Ki diferan montan de lajan miltip ki ta ka fè yon diferans nan lavi w? Ki chay espesifik montan sa yo ta soulaje?

*Ki lè pou bay lajan:*

- Si ou te kapab sèlman resevwa yon benefis lajan swa pandan gwosès la oswa nan mwa yo aprè gwosè la, kiyès nan de lè sa yo ki ta ede w plis? Poukisa?

*Frekans:*

- Eskè li pi bon pou resevwa yon benefis lajan angwo pandan gwosès la, oubyen ti peman regilye?

*Dire:*

- Si w ta resevwa yon benefis lajan pandan gwosès la nan yon tan limite, ki minimòm ki ta fè yon diferans enpòtan nan lavi w?

*Mekanis peman:*

- Sou ki fòm yon benefis lajan nou ta dwe bay pasyan ansent? Pa egzanp, kòm yon depo labank, yon transfè telefòn, pran nan men kesye, oubyen ki lòt jan ou ta vle fè l. Tanpri bay otan detay ke ou kapab.

*Kesyon final yo:*

- Ki laj ou genyen?
- Eskè w ansent oubyen aprè akouchman?
- Konbyen pitit ou genyen?
- Eskè gen yon lòt bagay ou ta renmen nou konnen?

Entèvyou a fini. Mèsi anpil pou patisipasyon ou!
